# Mapping the Distribution and Depth of Metacognitive Processes in Generative AI-Assisted Learning: Evidence from Interaction Logs and Concurrent Think-Aloud Protocols

**DOI:** 10.3390/bs16081365

**Published:** 2026-08-09

**Authors:** Shijin Li, Junjian Liu

**Affiliations:** 1 Jing Hengyi School of Education, Hangzhou Normal University, No. 2318, Yu Hang Tang Road, Hangzhou 311121, China; 2 Chinese Education Modernization Research Institute, Hangzhou Normal University, Hangzhou 311121, China

**Keywords:** generative AI, metacognitive processes, monitoring and control, human–AI interaction, think-aloud protocol, interaction logs

## Abstract

Generative artificial intelligence (GenAI) is increasingly used as a cognitive aid in education, yet no single process measure captures how learners monitor and control GenAI-assisted work. Guided by a reciprocal monitoring-control account of metacognition, this descriptive, exploratory study examined what interaction logs and concurrent think-aloud speech make observable during a 90 min instructional-design task. Seventy-nine undergraduates from 11 majors completed the task with DeepSeek support. The same two-dimensional rubric was applied separately to both records: each retained semantic unit received one primary operational category (planning, monitoring, or regulation) and one evidence-depth label (implicit, awareness, strategic, or reflective). Planning was treated as prospective control and regulation as adaptive control after evaluation or feedback. The interaction-log and think-aloud records yielded 274 and 193 retained instances, respectively. In both sources, regulation was the most frequent primary label and monitoring the least frequent, while evidence-depth ratings were concentrated at awareness and strategic. The combined strategic and reflective share was 25.4 percentage points higher in think-aloud than in interaction-log monitoring units and 9.9 points higher in think-aloud than in interaction-log planning units; for regulation, it was 14.0 points higher in interaction-log than in think-aloud units. These aggregate contrasts are descriptive because coded instances are nested within participants and the sources are paired within individuals. The central contribution is that verbalized monitoring information and enacted control leave different observable traces. Combining the sources therefore provides a fuller account of metacognitive activity than either record alone, without establishing monitoring accuracy, process effectiveness, or learning outcomes.

## 1. Introduction

Generative artificial intelligence (GenAI) is reshaping education by providing fluent content generation, personalized dialogue, and on-demand support (Kasneci et al., 2023). Its outputs are neither neutral nor uniformly reliable: GenAI systems can reproduce factual errors and social biases present in their training corpora. Productive use therefore depends not only on formulating requests but also on evaluating responses against task criteria and deciding whether and how to change course. These demands make learner monitoring and control central to the study of GenAI-assisted learning. Recent reviews have documented the rapid growth of GenAI applications and their pedagogical and ethical implications (Bond et al., 2024; Crompton & Burke, 2023; Tu & Lu, 2025), but the processes through which learners direct and evaluate GenAI remain under-described.

The cognitive demands involved in evaluating model output, judging task progress, and deciding whether and how to revise an approach are closely related to metacognition. Flavell (1976) introduced metacognition as knowledge and cognition about cognitive phenomena, and his later model described interactions among metacognitive knowledge, metacognitive experiences, cognitive goals, and cognitive actions (Flavell, 1979). Contemporary accounts commonly distinguish metacognitive knowledge from metacognitive processes (Brown, 1987; Nelson & Narens, 1990; Veenman et al., 2006). Metacognitive knowledge concerns knowledge about persons or the self, tasks, and strategies. Across learning contexts, metacognitive engagement has been associated with academic achievement (Broadbent & Poon, 2015) and identified as a potential leverage point for addressing learning gaps (Loeffler et al., 2019; Sáiz-Manzanares et al., 2024). Online metacognitive processes are commonly organized through reciprocal monitoring and control: monitoring supplies information about the current state, whereas control selects, initiates, modifies, or terminates subsequent activity. Within this architecture, planning is treated as prospective control, whereas regulation is used in the present coding scheme as a narrower operational label for adaptive changes made after evaluation or feedback. Cyclical models of self-regulated learning provide task-temporal context for goal setting, monitoring, and adaptive action (Pintrich, 2000; Zimmerman, 2000; Efklides, 2011), but the present study does not assess motivation, affect, goal orientation, or metacognitive knowledge. Its empirical focus is limited to observable evidence of metacognitive processes in interaction logs and concurrent verbal reports.

Recent GenAI interventions include scripted and reciprocal-teaching scaffolds grounded in ICAP (Hayashi et al., 2025), inquiry environments organized around teaching, cognitive, and social presence (Chen et al., 2024), visual analytics for learner-AI interaction (Yan et al., 2025), and reflective-writing assistants (Neshaei et al., 2025). These studies show how GenAI environments can prompt goal setting, evaluation, monitoring, and reflection. However, evaluations often emphasize intervention effects or aggregate outcomes (Chen et al., 2024; Martha et al., 2023) and provide less evidence about how learners externalize planning, monitoring, and adaptive control during interaction (Huang et al., 2025). The unresolved issue is therefore process visibility, not whether one operational category occurs more often than another.

Each online measure captures only part of that process. Think-aloud protocols provide time-proximal verbal evidence but can be reactive and omit unspoken activity (Eccles & Arsal, 2017; Ericsson & Simon, 1993). Interaction logs preserve enacted prompts and revisions but not silent evaluations. Coordinated online measures can therefore be compared without treating either channel as a criterion measure (Azevedo, 2020; Molenaar et al., 2023; Veenman et al., 2006). In GenAI-assisted work, a learner may evaluate an output silently, verbalize a planned revision without enacting it, or revise an artifact with little accompanying speech.

This study addresses that measurement gap by applying the same operational rubric separately to interaction logs and concurrent think-aloud speech. Its central contribution is to identify which forms of observable metacognitive evidence each source makes visible. Alignment describes patterns present in both records, whereas divergence identifies channel-conditional evidence; neither is treated as proof that one source is superior or as a formal test of convergent validity. The study does not assess metacognitive knowledge, monitoring accuracy, motivation, affect, process effectiveness, or learning outcomes. Frequencies and depth ratings are therefore interpreted as properties of retained evidence rather than measures of learner proficiency.

RQ1. How are retained process instances distributed across the operational categories of planning, monitoring, and regulation within each data source?

RQ2. How are the evidence-depth labels distributed within each operational process category and data source?

RQ3. Where do interaction logs and think-aloud records descriptively align or differ in their operational-category distributions and evidence-depth profiles?

## 2. Literature Review

**Figure 1:**
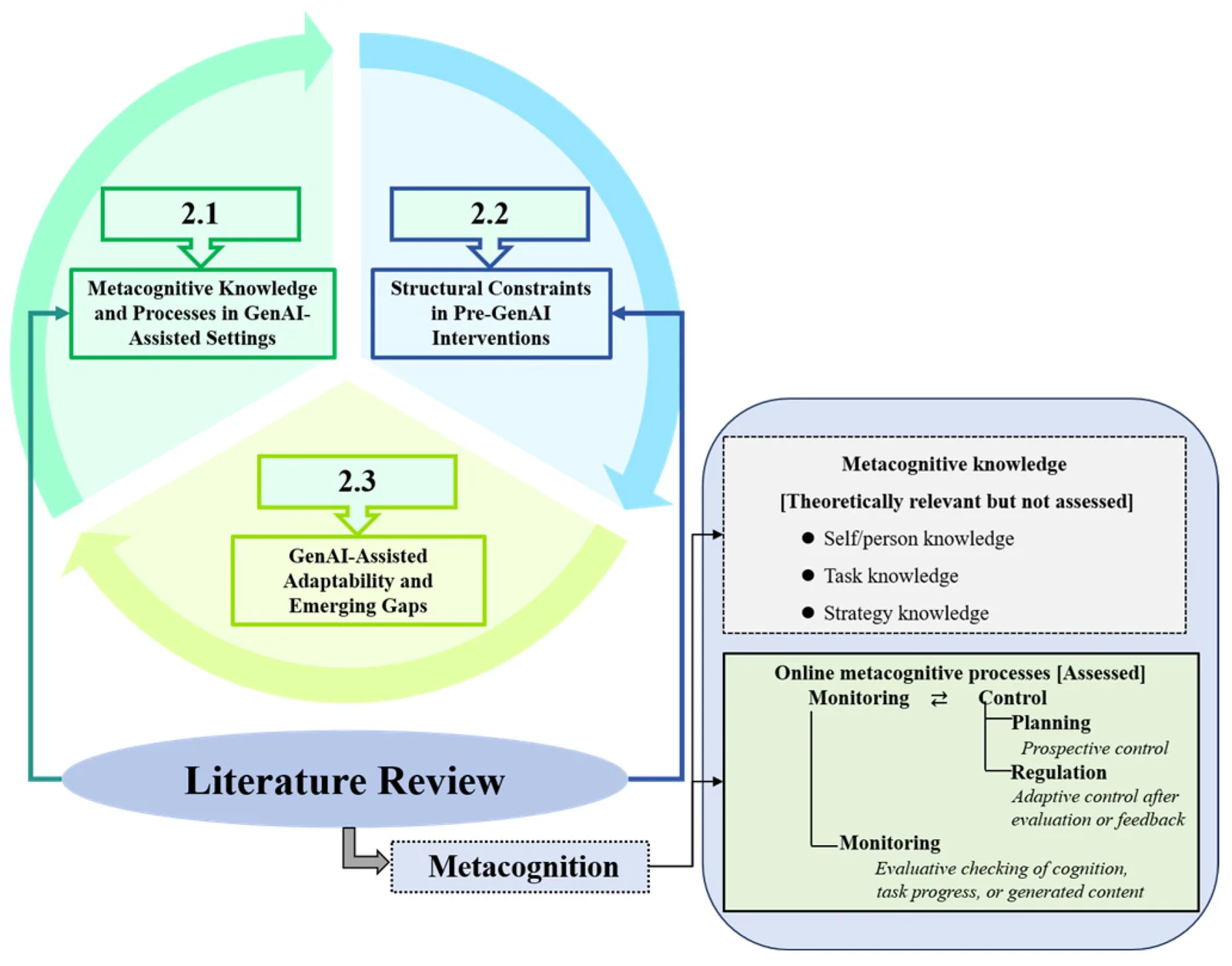
Conceptual and operational framework for metacognitive processes.

This review is organized around three connected dimensions. Section 2.1 first locates the study within contemporary models that distinguish metacognitive knowledge from metacognitive processes organized around a reciprocal monitoring–control architecture and then maps the observable processes to learner–GenAI interaction. Section 2.2 examines the structural limits of fixed and rule-based scaffolds and uses the Zone of Proximal Development to explain why support must be sufficient to enable performance without replacing learner control. Section 2.3 synthesizes the semantic adaptability and quasi-social cues introduced by generative systems while identifying unresolved questions about calibration, learner autonomy, and measurement-channel specificity. Figure 1 summarizes the conceptual and operational framework guiding the study.

### 2.1. Metacognitive Knowledge and Processes in GenAI-Assisted Settings

Metacognition encompasses two overarching components: metacognitive knowledge and metacognitive processes (Flavell, 1976, 1979; Brown, 1987; Nelson & Narens, 1990; Veenman et al., 2006). Metacognitive knowledge includes knowledge about the self or persons, tasks, and strategies. Metacognitive processes are organized around a reciprocal monitoring–control architecture: monitoring provides information about the current state of cognition, whereas control selects, initiates, modifies, or terminates subsequent cognitive activity. Developmental evidence indicates that these skills become increasingly differentiated and coordinated with age and experience, although their expression remains sensitive to task and domain demands (Veenman et al., 2006). The present study does not assess metacognitive knowledge and therefore makes no claims about learners’ knowledge of themselves, tasks, or strategies. It focuses on observable process evidence. Within the monitoring–control architecture, planning is conceptualized as a prospective form of control. For operational analysis, planning, monitoring, and regulation are coded separately to capture prospective organization, evaluative checking, and adaptive modification, respectively; the regulation label is reserved for observable changes to prompts, strategies, or artifacts after evaluation or feedback. This analytical separation preserves temporal and functional distinctions in the data and does not position the three categories as independent or coequal higher-order constructs.

The metacognitive agent in this account is unequivocally the learner, not the GenAI system. GenAI is treated as an external cognitive and communicative resource whose output may become an object of the learner’s monitoring and control. A prompt that specifies goals, constraints, or a sequence can externalize planning; a judgment that compares a model response with a curricular or task criterion can externalize monitoring; and a targeted revision made after that judgment can externalize regulation. In GenAI-assisted work, the monitored object may therefore extend from the learner’s internal cognition or individual draft to a co-produced artifact (Molenaar et al., 2023), but metacognitive agency remains with the learner. A fluent or sophisticated model response is not, by itself, evidence of sophisticated learner metacognition. This boundary prevents properties of the generated output from being misclassified as learner processes.

The frameworks used in this study serve distinct, non-interchangeable roles at different analytic levels. Nelson and Narens’ (1990) monitoring–control architecture provides the core metacognitive structure: monitoring supplies information about the current state, whereas control changes subsequent cognitive activity. Brown (1987) and cyclical models of SRL (Pintrich, 2000; Zimmerman, 2000) provide conceptual and task-temporal context for prospective planning, ongoing monitoring, and adaptive action. However, the present study does not assess the broader motivational or affective components of SRL. The Zone of Proximal Development (Vygotsky, 1978) is used only to interpret whether scaffolding preserves learner responsibility and is appropriately calibrated to learner need; it does not determine the coding categories. Perkins (1995) and Adinda et al. (2023) inform the labels and boundaries of the evidence-depth rubric, whereas studies of GenAI-assisted learning inform task-specific operational examples. These frameworks are therefore not combined as interchangeable accounts of metacognition. The monitoring–control architecture defines the focal process relation, while the other sources provide theoretical context, operational guidance, or interpretive support.

No single online measure captures all of these processes. Logs preserve enacted requests and revisions but not unexpressed evaluations. Think-aloud data preserve some concurrent evaluations and intentions but may omit tacit activity and may include speech prompted by the protocol. Using a shared coding framework makes the channels comparable at the level of observable evidence while preserving their different affordances. The analytic goal is therefore complementarity rather than interchangeable measurement or validation of one source by the other.

### 2.2. Structural Constraints in Pre-GenAI Interventions

Before generative models, metacognitive interventions commonly used fixed prompts or rule-based adaptive support. Fixed prompts delivered scheduled cues; rule-based systems triggered feedback when predefined trace indicators crossed a threshold (Dever et al., 2024; Munshi et al., 2023; Vangsness & Young, 2021). These approaches can make target strategies explicit and can support initial acquisition, but their adaptation is bounded by the rules specified in advance. As a result, the system’s response may not address the semantic content of a learner’s concern or the criterion underlying a judgment.

The literature also identifies a support dilemma. Prompts that are too frequent, directive, or poorly timed may interrupt task processing or displace learner control, whereas support that is too sparse may fail to elicit monitoring or leave a strategy inaccessible (Schumacher & Ifenthaler, 2021; Vangsness & Young, 2021). The Zone of Proximal Development (ZPD) provides an educational framework for this dilemma: scaffolding should enable performance beyond what the learner can currently accomplish independently without transferring responsibility for monitoring and control to the support system (Vygotsky, 1978). Within this lens, appropriately calibrated support can create occasions for learners to diagnose difficulty, evaluate assistance, and adjust strategy. GenAI may enable more context-sensitive assistance, but generativity does not itself establish that support is appropriately timed, sufficiently contingent, or matched to learner need. Calibration concerns both the accuracy of the learner’s judgment and the fit between assistance and the learner’s current state (Fleming & Lau, 2014). Neither form of calibration was directly measured in the present study.

### 2.3. GenAI-Assisted Adaptability and Emerging Gaps

GenAI extends earlier scaffolds through open-ended language generation and sensitivity to conversational context. It can respond to unstructured goals, generate alternative representations, and prompt explanation or reflection (Chen et al., 2024; Neshaei et al., 2025; Wu et al., 2025). At the same time, digital technologies can recruit conceptual and social processing, including anthropomorphic attribution, that differs from interaction with mechanical tools (Federico et al., 2025, 2026). These properties make GenAI both an external cognitive resource and a quasi-social information source. Learners must therefore monitor not only task progress but also the reliability of their own judgments and of generated information, a concern aligned with work on metacognition in contested information environments (Fischer et al., 2023).

The evidence nevertheless remains mixed. Viewed through the ZPD, the educational value of GenAI support depends on whether assistance extends learners’ current capabilities while preserving responsibility for monitoring and control. Appropriately calibrated scaffolding may support engagement, whereas overly directive assistance may shift planning, evaluation, and revision from learner regulation to system delegation (Kong & Yang, 2024; Zhan & Yan, 2026). Adaptive or fluent assistance should therefore not be equated with metacognitive benefit.

Three complementary lines of research clarify the remaining measurement gap. First, reviews of self-regulated learning and AI demonstrate the value of coordinated multimodal and multichannel process measures but do not provide a shared GenAI-specific rubric for comparing observable metacognitive evidence (Molenaar et al., 2023). Second, conceptual and human–computer interaction research identifies the monitoring and control demands created by GenAI but rarely compares verbalized evaluations with enacted prompts and revisions (Tankelevitch et al., 2024). Third, intervention studies involving inquiry agents, reflective-writing assistants, and visual learning analytics demonstrate several forms of adaptive scaffolding, yet they rely on heterogeneous outcomes and provide limited fine-grained evidence about observable learner processes within structured tasks (Chen et al., 2024; Neshaei et al., 2025; Yan et al., 2025). Related neurocognitive and theoretical research highlights conceptual and social processing, including anthropomorphic attribution, but its relationship with educational metacognitive traces and support calibration remains insufficiently tested (Federico et al., 2025, 2026). Collectively, these studies do not use a common coding framework to compare concurrent verbalized cognition with enacted interaction traces. The present study addresses this measurement gap descriptively by applying the same operational rubric to interaction logs and think-aloud protocols. It does not test whether GenAI improves metacognition, whether support falls within an individual learner’s ZPD, whether monitoring is accurate, or whether observed process depth predicts product quality.

## 3. Method

### 3.1. Research Context and GenAI Interaction Workflow

The study took place in an undergraduate course at the University of S in a technology-enhanced learning environment. The curriculum required students to integrate learner analysis, measurable objectives, task and content analysis, instructional activities and strategies, learning resources and environment, assessment and evaluation, and reflective alignment. All participants used DeepSeek-V3, as identified in the system records, during the 90 min instructional-design task and remained responsible for the final plan. Beyond the common task instructions provided in Appendix B, no standardized user prompt was imposed. Participants independently formulated and revised their prompts; consequently, prompt content and interaction sequences varied across participants. The interaction logs preserved these participant-generated user inputs.

From the participant’s perspective, interaction with the platform involved three recurring components. These components describe the practical workflow and interface use rather than theoretical stages or coding categories.

(1) Task specification and prompt entry.

Participants entered requests in the platform’s text-input field. They could provide the instructional context, identify required components, specify constraints, ask questions, or request assistance with a selected aspect of the developing plan.

(2) Model-response presentation.

DeepSeek displayed generated responses in the conversational interface. Participants could read the response while referring to the task requirements and their developing instructional-design artifact. They were not required to accept or incorporate the generated content.

(3) Follow-up interaction and artifact revision.

Participants could continue the dialogue by requesting clarification, correction, elaboration, alternatives, or further assistance. They could incorporate, reject, or modify generated content while revising their instructional-design plan. The interaction could return repeatedly to prompt entry, response review, and follow-up dialogue during the 90 min session.

These components constituted a recurrent workflow and were not treated as direct indicators of particular metacognitive processes. During the task, screen recording captured the visible interaction sequence, while synchronized audio recording captured participants’ concurrent verbalizations. Data preparation and coding were conducted after task completion and are described in Section 3.3.

### 3.2. Participants

The study involved 79 consenting undergraduates from 11 majors at the University of S (M age = 20.1 years, SD = 0.86). The convenience sample included students from 11 majors, but the disciplinary distribution was uneven: 45 participants majored in Preschool Education (57.0%) and 14 in Primary Education (17.7%), together accounting for 74.7% of the sample. The remaining participants were drawn from Ideological and Political Education, Chinese Language and Literature, Nursing, Biological Sciences, English Teacher Education, Public Administration, Translation, History, and Physics.

To characterize participants’ self-reported GenAI background, a pre-task survey assessed prior experience with and attitudes toward GenAI tools (Table 1). Prior exposure varied: 20.2% had never used GenAI, 34.2% had used it but remained unfamiliar with it, 32.9% reported moderate familiarity, and 12.7% reported relative familiarity. Attitudes were generally favorable: 64.5% were somewhat supportive and 12.7% were strongly supportive; 20.2% were neutral; and 1.3% were somewhat opposed and 1.3% were strongly opposed. These responses indicate variation in self-reported exposure and acceptance; they do not constitute validated measures of AI literacy, technical proficiency, or GenAI competence and therefore are not used to infer participants’ ability to work effectively with GenAI. Informed-consent procedures and the complete pre-task survey are provided in Appendix A.

### 3.3. Data Collection and Coding

The study used two process-data streams—screen-recorded interaction behavior and concurrent think-aloud speech—in a single-group, task-centric design. No instructional condition was manipulated. Each participant completed one cohesive project during a 90 min session. The task instructions in Appendix B explicitly shaped expected behavior and are treated as a source of demand characteristics.

Before the task, participants completed a standardized 10 min think-aloud training period that included an explanation and researcher modeling, followed by a 5 min warm-up exercise. During the task, participants were asked to verbalize concurrent thoughts; the researcher used the neutral prompt “Please keep talking” only after more than 5 s of silence. Participants were also instructed to read AI responses aloud. The archived processing record does not establish whether verbatim model text was systematically removed before coding. Consequently, not all retained verbal content can be assumed to constitute independent evidence of learner cognition.

#### 3.3.1. Two-Dimensional Coding of Observable Metacognitive Processes

Each retained semantic unit was coded independently along two dimensions. Dimension 1, operational process category, answered what metacognitive function was observable and assigned planning, monitoring, or regulation. Dimension 2, depth of explicit metacognitive evidence, answered how explicitly and integratively the unit articulated a goal, criterion, procedure, evaluation, or transfer and assigned implicit, awareness, strategic, or reflective. The categories describe evidence contained in a prompt or utterance; they do not attribute metacognition to the AI response itself.

(1) Dimension 1: Operational Metacognitive Process Category

Flavell’s early account distinguished metacognitive knowledge from metacognitive experiences and described their relations with cognitive goals and actions (Flavell, 1976, 1979). Contemporary accounts distinguish metacognitive knowledge—knowledge about the self or persons, tasks, and strategies—from metacognitive processes organized through reciprocal monitoring and control (Nelson & Narens, 1990; Veenman et al., 2006). The present study did not assess metacognitive knowledge or affective experience. It focused on observable evidence of metacognitive processes in learner prompts and concurrent speech. Within the monitoring–control architecture, planning was treated as prospective control, monitoring as information about a current cognitive or task state, and regulation as an observable modification of ongoing action after evaluation or feedback.

The operational descriptions were informed by regulatory activities discussed in the broader self-regulated learning literature (Pintrich, 2000) and were adapted to the present GenAI task. The three operational labels were assigned mutually exclusively according to the primary function of a semantic unit. Prospective organization or a constraint was coded as planning; an evaluation or status judgment without an enacted change was coded as monitoring; and a change made after feedback or evaluation was coded as regulation. When a unit contained both a judgment and a change, it was segmented if each function formed an independently meaningful clause; otherwise, the culminating function determined the category. Table 2 provides the decision rule and observable example for each category.

(2) Dimension 2: Depth of Explicit Metacognitive Evidence

The depth dimension was independent of the operational process category because RQ2 examines evidence-depth distributions within each operational category and source, while RQ3 examines descriptive cross-source differences in those profiles. Depth is a property of coded evidence, not a classification of learners. It was defined as an ordinal increase in the explicitness and integration of metacognitive evidence contained in a unit, not as a second taxonomy of metacognitive processes and not as a direct measure of latent proficiency, monitoring accuracy, language ability, AI literacy, task knowledge, writing quality, or prompt sophistication. Awareness and reflective therefore did not map specifically to monitoring: planning, monitoring, and regulation units could each receive any of the four evidence-depth ratings when their wording met the corresponding decision rule.

The four evidence-depth labels—implicit, awareness, strategic, and reflective—were retained for continuity with the coded dataset (Table 3). Their initial formulation drew on Perkins’ (1995) four-level account and Adinda et al.’s (2023) analysis of questioning characteristics, while the task-specific examples were informed by research on GenAI interaction (Chen et al., 2024; Tankelevitch et al., 2024). In the present task-adapted rubric, the labels are ordered only with respect to the explicitness and integration of evidence observable within a semantic unit. These sources informed the rubric labels, boundaries, and examples but were not treated as evidence for a validated developmental or proficiency scale; accordingly, the rubric classifies the evidence expressed in a semantic unit rather than the learner.

Four boundary rules guided ambiguous cases. First, “Is this objective appropriate?” is monitoring at awareness depth because it states a concern without a criterion; “Check whether the objective is measurable using an observable-performance criterion” is monitoring at strategic depth. Second, a multi-part prompt is not automatically strategic: a list copied from the task sheet without a decision rule remains awareness or underspecified evidence. Third, “Revise the assessment” is regulation at awareness depth, whereas a revision tied to a stated alignment criterion is regulation at strategic depth. Fourth, evidence is reflective only when the learner evaluates the effectiveness or limitation of an approach and uses that evaluation to justify adaptation or transfer. If monitoring and adjustment are independently meaningful, the clauses are segmented; otherwise, the culminating function determines the operational category.

#### 3.3.2. Think-Aloud Data Coding Rules

Following qualitative content-analysis procedures used in prior think-aloud research (Halmo et al., 2024; Xu & Zhu, 2024), retained verbal segments were coded with the same process-category and evidence-depth rules as interaction-log units. Conceptual symmetry did not make the channels equivalent: a verbalized intention was evidence in the think-aloud channel, whereas an enacted prompt or revision was evidence in the interaction-log channel. Verbatim reading of model output, if retained in the archived transcript, could reproduce model language without establishing learner evaluation. Because the archived processing record does not establish whether such reading was systematically removed, this issue is treated as a limitation rather than classified as learner cognition.

### 3.4. Data Analysis

The study used a coordinated multi-source analytic workflow informed by process-oriented approaches to combining behavioral and verbal evidence (Laudenbach et al., 2024). Interaction logs and concurrent think-aloud speech were segmented and coded separately, summarized within source, and then compared descriptively for alignment and divergence. This design supports complementary observation of enacted and verbalized evidence; it does not establish interchangeability, criterion validity, or superiority of either channel.

#### 3.4.1. Data Preprocessing and Reliability Verification

Unitization preceded category assignment. For interaction logs, a semantic unit was a prompt–response exchange or separable clause serving one metacognitive function; phatic communication, off-task material, and system-latency errors were excluded. For think-aloud data, a unit was a purposeful utterance or clause that expressed a goal, judgment, procedure, or adjustment; non-lexical fillers and task-irrelevant speech were excluded. The archived record does not report the total number of raw verbal units or a unit-by-unit exclusion ledger and does not document whether verbatim model reading was removed. These gaps prevent a complete retained-versus-excluded accounting for the verbal channel.

Two trained researchers independently coded a random 20% subsample. Cohen’s κ was calculated separately for process-category and evidence-depth assignment within each source: process category—interaction logs κ = 0.90 and think-aloud κ = 0.87; evidence depth—interaction logs κ = 0.86 and think-aloud κ = 0.85. Under the conventional benchmarks of Landis and Koch (1977), these values fall within the almost-perfect agreement range. Disagreements were resolved by consensus with a third senior researcher. However, separate unitization reliability, confidence intervals for κ, the exact double-coded sample size, and participant-level coder records are unavailable. The coefficients therefore support consistency of category assignment only, not reliability of semantic-unit boundaries.

#### 3.4.2. Coding of Interaction-Log Data

The interaction-log corpus yielded 274 retained process instances from 79 participants (3.47 retained instances per participant on average). Dimension 1 classified each unit as planning, monitoring, or regulation. Dimension 2 rated explicit evidence as implicit, awareness, strategic, or reflective.

#### 3.4.3. Coding of Think-Aloud Data

The think-aloud corpus was managed in NVivo 12 and yielded 193 retained process instances from 79 participants (M = 2.44 retained instances per participant). The same two-dimensional rubric was applied, but codes referred to concurrent verbal evidence rather than enacted interaction.

#### 3.4.4. Statistical Analysis

Descriptive summaries were produced in SPSS 27.0, while the think-aloud corpus was managed in NVivo 12. Counts and within-source percentages summarize the three operational process categories and four evidence-depth labels. Category-level Pearson correlations were not used because correlations across three or four compositional proportions are not meaningful convergence tests. Pooled chi-square tests were also omitted because coded instances are nested within participants and the two sources are paired within individuals. The archived participant-by-category matrix needed for paired analyses or mixed-effects models is unavailable. Cross-source differences are therefore reported only as descriptive percentage-point contrasts among retained instances. Confidence intervals for these contrasts are not reported because intervals calculated from pooled units would incorrectly assume independence; valid participant-level intervals cannot be reconstructed from aggregate counts.

## 4. Results

### 4.1. Distribution of Observable Metacognitive Process Categories

**Figure 2:**
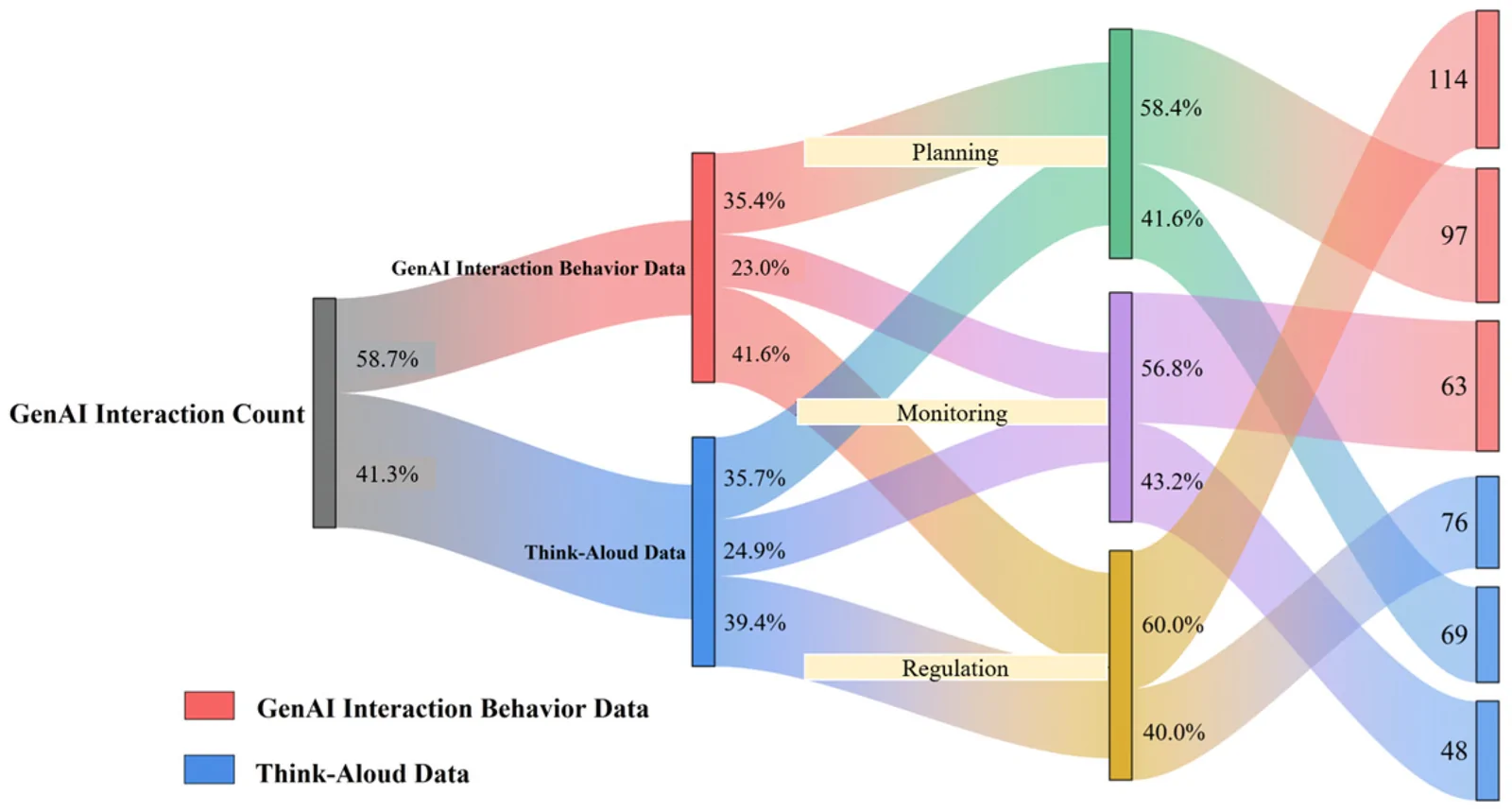
Descriptive distribution of retained observable metacognitive process instances by data source in the GenAI-assisted task.

Across the two separately coded sources, 274 interaction-log instances and 193 think-aloud instances met the retention criteria, as illustrated in Figure 2. These totals are pooled counts of retained semantic units, not independent observations and not estimates of the number of learners who enacted a process. The following sections therefore report descriptive counts and percentages for the operational process categories within each source. Differences in retained-instance totals may reflect channel affordances, protocol effects, unitization decisions, and task instructions; they are not evidence that one source captures metacognition more accurately.

#### 4.1.1. GenAI Interaction-Log Results

**Figure 3:**
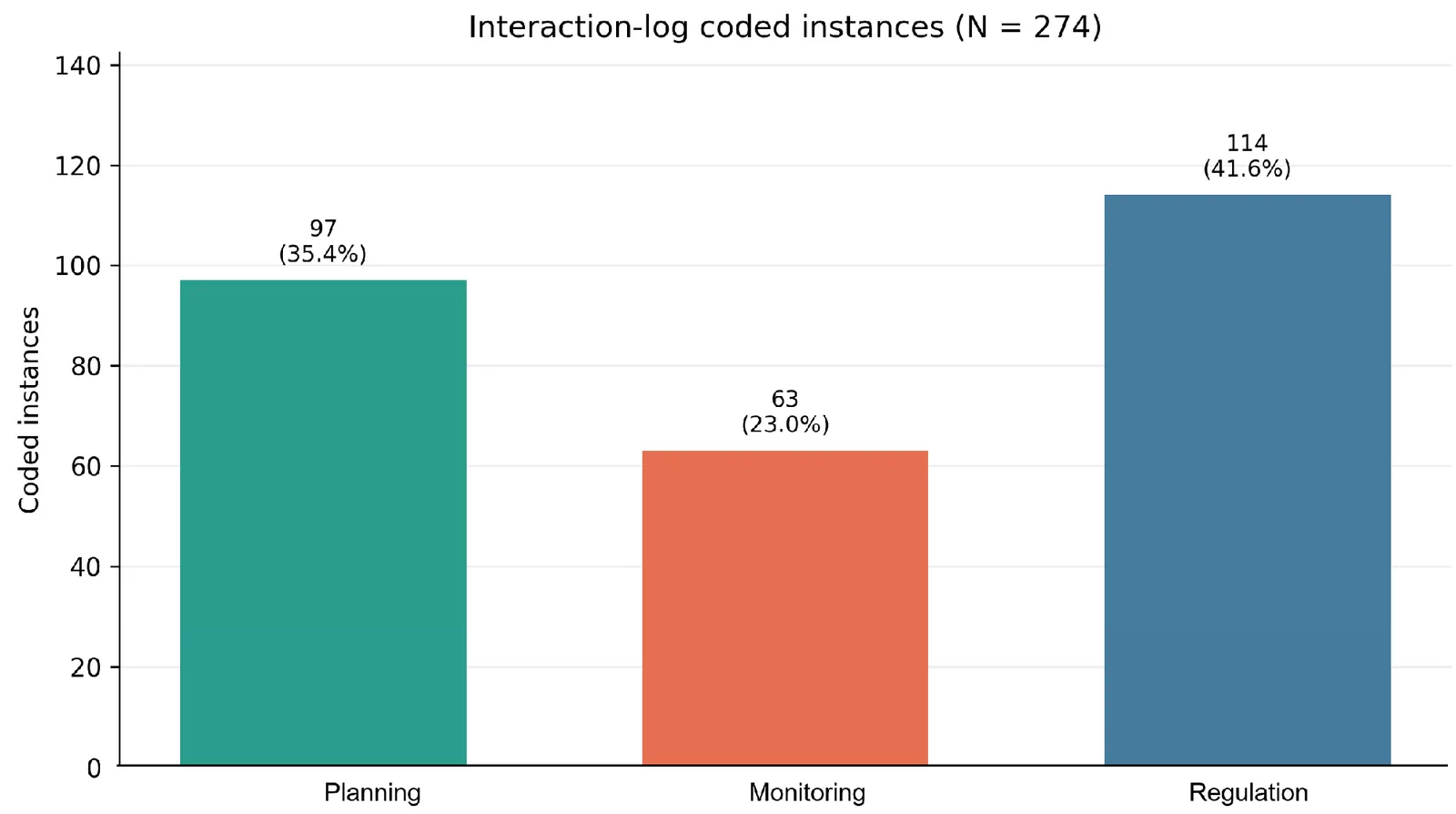
Descriptive distribution of operational metacognitive process categories in the interaction-log record.

The interaction logs contained 274 retained instances, an average of 3.47 per participant. As shown in Figure 3, regulation accounted for 114 instances (41.6%), planning for 97 (35.4%), and monitoring for 63 (23.0%). These percentages describe the coded record from a task that explicitly encouraged planning and revision.

#### 4.1.2. Think-Aloud Results

**Figure 4:**
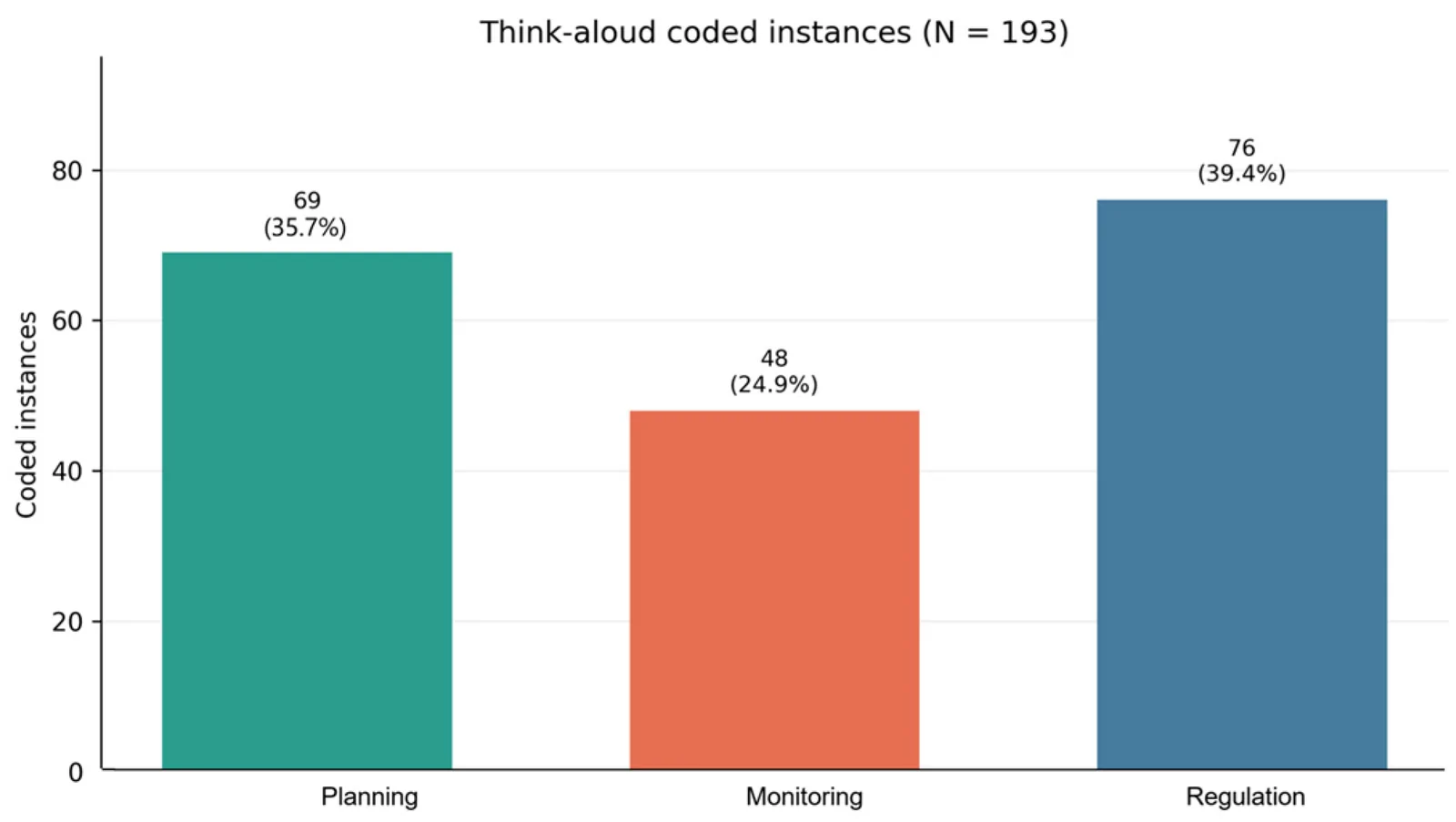
Descriptive distribution of operational metacognitive process categories in the think-aloud record.

The think-aloud record contained 193 retained instances (M = 2.44 per participant). As shown in Figure 4, regulation accounted for 76 instances (39.4%), planning for 69 (35.7%), and monitoring for 48 (24.9%). The ordering resembles the interaction-log record at the pooled level, but this resemblance is descriptive. The lower relative frequency of monitoring is a descriptive property of the retained corpus and does not establish monitoring accuracy, trust calibration, or consequences for product quality.

#### 4.1.3. Cross-Source Descriptive Alignment

The pooled category profiles were similar: regulation and planning together represented 77.0% of interaction-log instances and 75.1% of think-aloud instances, while monitoring represented 23.0% and 24.9%, respectively. Neither a correlation nor a significance test was used because three compositional proportions that necessarily sum to one cannot provide a valid inferential convergence test. Similar aggregate shares may also mask participant-level or temporal differences. This result is therefore reported only as descriptive alignment in the archived pooled counts.

### 4.2. Variation in the Depth of Explicit Metacognitive Evidence Across Data Sources

**Figure 5:**
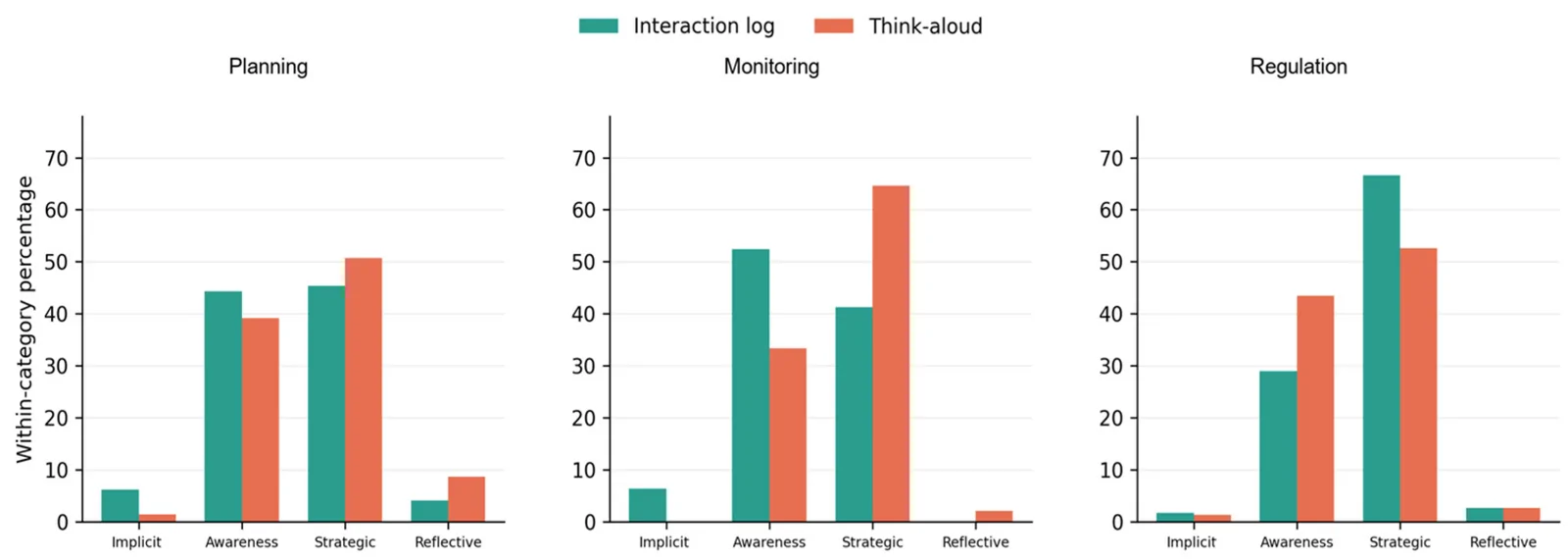
Descriptive depth distributions by operational metacognitive process category and data source.

Evidence-depth ratings in both sources were concentrated under the awareness and strategic labels, whereas implicit and reflective evidence was uncommon (Figure 5). This pattern is described simply as a concentration in the two middle rubric categories. Because depth refers to explicit evidence in a coded unit, it should not be read as a direct distribution of learners’ latent metacognitive ability.

Table 4 reports the underlying counts and within-category percentages. The final column presents descriptive percentage-point contrasts in the combined strategic and reflective share. These contrasts summarize retained instances only and do not adjust for nesting or within-participant pairing.

#### 4.2.1. Planning: Cross-Source Depth Description

Across both sources, 166 planning instances were retained (97 interaction logs; 69 think-aloud). Awareness and strategic evidence accounted for most instances. Higher-depth evidence (strategic plus reflective) represented 49.5% of interaction-log instances and 59.4% of think-aloud instances, or 9.9 percentage points higher in the think-aloud record. This contrast may reflect verbalized intentions that were not enacted, but the available data do not identify its mechanism.

The pattern is consistent with channel complementarity: think-aloud speech can contain prospective decomposition or constraints that do not appear in the next prompt, while an interaction log can contain concise enacted planning whose rationale is not spoken. Without time-linked participant-level sequences, these possibilities cannot be separated or used to evaluate planning quality.

#### 4.2.2. Monitoring: Cross-Source Depth Description

The archive contained 111 monitoring instances (63 interaction logs; 48 think-aloud). Higher-depth evidence represented 41.3% of interaction-log instances and 66.7% of think-aloud instances, or 25.4 percentage points higher in the think-aloud record. Reflective monitoring was rare in both sources. This result shows that explicit evaluative criteria appeared more often in retained verbal monitoring units than in retained log units; it does not show that learners trusted the model, that their judgments were inaccurate, or that greater monitoring would improve outcomes.

One measurement account is that learners can verbalize an evaluation without translating it into a prompt. Other possibilities include think-aloud reactivity, unitization differences, or contamination by verbatim reading of model text. Because calibration, trust, and cognitive load were not measured, no psychological mechanism is selected among these alternatives.

#### 4.2.3. Regulation: Cross-Source Depth Description

The archive contained 190 regulation instances (114 interaction logs; 76 think-aloud). Higher-depth evidence represented 69.3% of interaction-log instances and 55.3% of think-aloud instances, or 14.0 percentage points higher in the interaction-log record. The concentration of strategic evidence means that many retained revisions were tied to an articulated task feature; it does not establish that a revision was effective or improved the final design.

Enacted adjustment was more explicit in the interaction-log record than in concurrent speech. GenAI editing affordances, reduced verbalization during action, task instructions, and coding-unit differences are possible explanations. Because these factors were not independently measured or manipulated, the contrast is reported without a causal or efficiency interpretation.

## 5. Discussion

This study descriptively mapped retained instances of observable metacognitive processes during a GenAI-assisted instructional-design task, using interaction logs and concurrent think-aloud speech as complementary but non-equivalent records. Two observations organize the discussion. First, the frequencies of the mutually exclusive operational labels differed within each record. Second, the depth of explicit metacognitive evidence varied by operational category and data source rather than following a uniform cross-source pattern. These findings concern coded semantic units observed within a structured instructional-design task. The central contribution is therefore methodological and descriptive: the two complementary records yielded category-specific evidence profiles that could not have been identified from either source alone.

### 5.1. Distribution of Operational Process Categories

Within the reciprocal monitoring-control architecture, planning and regulation are both manifestations of control rather than independent theoretical components: planning was operationalized as prospective control, whereas regulation was operationalized as adaptive control following evaluation or feedback (Nelson & Narens, 1990; Veenman et al., 2006). Monitoring was operationalized as evaluative checking of the learner’s current cognitive or task state or of the developing artifact. Accordingly, the present frequencies compare mutually exclusive coding labels assigned according to the primary observable function of each retained semantic unit; they do not compare independent components of metacognition. Among these labels, regulation was most frequent and monitoring least frequent in both records. In the interaction-log record, regulation accounted for 41.6% of retained instances, planning for 35.4%, and monitoring for 23.0%. In the think-aloud record, the corresponding percentages were 39.4%, 35.7%, and 24.9%. This empirical ordering does not imply that regulation is theoretically independent of, or dominant over, planning or monitoring.

The comparatively smaller share of units coded primarily as monitoring resembles reports of limited monitoring in some non-AI settings (Zhang et al., 2022), but direct equivalence cannot be assumed because tasks, coding rules, and units of analysis differ. More importantly, the present study included neither a non-GenAI comparison nor a theory-based reference distribution. It therefore cannot show that GenAI failed to remedy a monitoring deficit or that the observed monitoring frequency was insufficient. The task explicitly encouraged planning and revision, and the platform enabled rapid generation and modification of content (Yan et al., 2024); both features may have shaped which functions became visible in the records. Although regulation without adequate antecedent monitoring has been described as inefficient trial and error in non-AI contexts (Ali & Yasmeen, 2019), the aggregate counts do not preserve the temporal evidence needed to determine whether any coded regulation instance lacked prior monitoring.

These observations motivate a reciprocal monitoring-control scaffold as a design hypothesis, not a fixed sequence or an established recommendation. Such a scaffold could prompt learners to externalize a goal or evaluative criterion as prospective control, compare the current AI response or developing artifact with that criterion, select and justify an adaptation when a discrepancy is identified, and monitor the resulting state. New information could return the learner to any function in the cycle. The testable proposition is that making the informational relation between monitoring and control explicit may change the quality and coordination of observable process evidence. Whether such scaffolding improves learning or instructional-design quality must be examined in controlled studies that compare alternative scaffolds and include independently assessed outcomes.

### 5.2. Cross-Source Variation in the Depth of Explicit Metacognitive Evidence

Within each operational process category, most retained units were coded at the awareness or strategic evidence-depth labels, whereas implicit and reflective evidence was comparatively uncommon. This dimension was informed by Perkins (1995) and Adinda et al. (2023) but is used here only as an ordinal rubric for the explicitness and integration of evidence in a semantic unit. It is not a developmental sequence, a measure of learner proficiency, or a second taxonomy of metacognitive processes. In this rubric, implicit is operational shorthand for underspecified evidence in the semantic unit; it does not imply unconscious cognition or an absence of awareness. Implicit and reflective evidence are not proposed as qualitatively distinct latent mental states. Rather, they mark opposite observable endpoints of the rubric: the former contains no articulated metacognitive criterion, rationale, procedure, evaluation, or transfer, whereas the latter evaluates an approach’s effectiveness or limitation and uses that evaluation to justify adaptation or transfer. The absence of such explicit wording does not demonstrate that the learner lacked awareness.

The combined strategic and reflective share showed different descriptive contrasts across operational categories. For planning, it was 49.5% in interaction-log instances and 59.4% in think-aloud instances, 9.9 percentage points higher in the think-aloud record. For monitoring, it was 41.3% and 66.7%, respectively, 25.4 percentage points higher in the think-aloud record. For regulation, it was 69.3% in interaction-log instances and 55.3% in think-aloud instances, 14.0 percentage points higher in the interaction-log record. Thus, the direction and magnitude of the cross-source contrast depended on the operational category, with the largest observed contrast occurring for monitoring. These contrasts summarize pooled retained instances within each source and operational category. They are not participant-level estimates. No inferential test is reported because the archived data do not contain the participant-level matrix required to account for the nesting of instances and the within-participant pairing of the two records.

Several non-exclusive explanations remain possible. Think-aloud speech can make an evaluative criterion or prospective intention explicit even when it is not carried into the next prompt, whereas interaction logs can preserve concise enacted changes whose rationale is not verbalized. Monitoring large volumes of AI-generated content may also impose cognitive demands (Tankelevitch et al., 2024), and concurrent verbalization can alter or incompletely capture ongoing processing (De Bruin et al., 2020). Conversely, GenAI affordances can make revision actions especially visible in the interaction record. However, the observed contrasts could also reflect think-aloud reactivity, differences in unitization, the task instructions, or verbatim reading of model output. Because the archived processing record does not establish whether verbatim model text was systematically removed, the verbal corpus cannot be treated as exclusively learner-generated cognition. Cognitive load and the other proposed mechanisms were not measured, so none can be selected as the explanation for the source differences.

The study also cannot determine whether a greater frequency or depth of explicit monitoring evidence would improve the final artifact. That question requires outcome-linked evidence, such as independent ratings of instructional-design quality, together with time-linked participant-level process data. The present descriptive findings therefore motivate tests of reciprocal monitoring-control scaffolds, examination of how task and interface affordances shape enacted and verbalized evidence, and direct measurement of cognitive load; they do not establish that one data source or one operational category is superior.

### 5.3. Practical Implications for Educational Practice and GenAI-Supported Learning Environments

The channel-specific differences have practical implications for how educators interpret process evidence. Among the retained units, strategic and reflective evidence was more prominent in think-aloud planning and monitoring, whereas interaction logs contained a greater share of such evidence for regulation. These contrasts do not establish source superiority. Rather, they illustrate that interaction logs can preserve enacted revisions without their underlying rationales, whereas think-aloud speech can preserve evaluations or intentions that are not subsequently enacted. The absence of evidence in one channel should therefore not be taken as evidence that the corresponding process did not occur. Where feasible and ethically appropriate, educators could combine low-burden interaction traces with brief learner-authored checkpoints to improve formative visibility, not to enable surveillance, automated proficiency scoring, or validation of one source against the other.

At the task and interface levels, GenAI-supported learning spaces could invite learners to state a goal or evaluative criterion before generation, record a concise judgment after inspecting an output, and connect any subsequent revision to that judgment. These checkpoints could make the link between monitoring and control more visible while leaving learners responsible for accepting, rejecting, or revising model output. In a ZPD-informed design, the frequency and specificity of such prompts could be adjusted or gradually faded as learners demonstrate that they can articulate and apply their own criteria. Because the present task explicitly encouraged planning and revision, these features should be regarded as design propositions rather than evidence-based prescriptions derived from the observed frequency profile.

For formative feedback, the relevant signal is the relationship among temporally linked events rather than the pooled prevalence of any single label. When a revision is visible in the interaction log but its rationale is not articulated, an instructor or interface might invite the learner to explain the basis for the change. Conversely, when a discrepancy is verbalized but not followed by action, the learner might be prompted to decide whether adaptation is warranted. Any such support should be based on participant-level sequences and a coding scheme validated for the relevant task, learner population, and GenAI system. Controlled studies incorporating independent measures of learning and product quality are needed to determine whether these features improve monitoring–control coordination while preserving learner agency.

## 6. Conclusions, Limitations, and Future Directions

This study mapped observable metacognitive process evidence during a GenAI-assisted instructional-design task using screen-recorded interaction sequences and concurrent think-aloud speech. Among mutually exclusive operational labels assigned according to the primary function of retained semantic units, regulation was most frequent and monitoring least frequent in both records. This is an empirical ordering of coding labels, not a ranking of independent theoretical components: planning represents prospective control, whereas regulation represents adaptive control after evaluation or feedback. Evidence-depth ratings were concentrated at the awareness and strategic labels, while implicit and reflective evidence was comparatively uncommon. Descriptive cross-source contrasts in the combined strategic and reflective share varied by operational category: the think-aloud record was higher for planning and monitoring, whereas the interaction-log record was higher for regulation. These aggregate contrasts do not establish participant-level differences, channel superiority, or the quality of the underlying metacognitive processes. Methodologically, applying a shared rubric to complementary but non-equivalent records advances metacognitive process research by distinguishing verbalized judgments from enacted control and by revealing evidence that either source alone would leave unobserved. These channel-specific evidence profiles motivate, but do not establish, the use of complementary trace displays and reciprocal monitoring–control checkpoints as formative design hypotheses. Their effects on learner agency, process quality, and learning outcomes require outcome-linked experimental evaluation.

Several limitations bound these conclusions. First, the study used an observational, single-group design without a control condition, learning-outcome measure, or product-quality measure; causal and efficacy claims are therefore unwarranted. Second, coded instances were nested within participants and the two sources were paired within individuals, but the archived participant-by-category matrix was unavailable. Cross-source results are consequently limited to descriptive percentage-point contrasts among retained instances. Third, concurrent verbalization may have changed participants’ processing, and the instruction to read AI responses aloud creates a possible source of model-language contamination because the archived record does not establish whether verbatim reading was systematically removed. Fourth, the evidence-depth rubric is a task-adapted coding device whose construct validity as a general scale has not been established; it classifies explicit evidence in units and cannot identify learners’ latent ability, monitoring accuracy, or metacognitive knowledge. Fifth, the sample was drawn from one institution, one course, and one instructional-design task. The survey items provided descriptive information about prior experience and attitudes but were not validated measures of AI literacy or competence. The findings should therefore not be generalized to other disciplines, institutions, age groups, task types, or learners with different levels of GenAI literacy, trust, resistance, or acceptance without direct replication. The sampling composition further limits transferability. Participants were recruited from a single course at one institution, and 59 of the 79 participants majored in Preschool Education or Primary Education. The cohort also reported generally favorable attitudes toward GenAI, so the findings may not transfer to learners from other disciplines or with lower acceptance of GenAI. Although interaction and verbal traces were coded as observable process evidence, the study did not independently assess the quality of those processes, learning progress, achievement, or instructional-design quality. The observed evidence profiles therefore cannot be linked to learning effectiveness.

Future research should first link time-resolved process evidence to independently assessed learning and design outcomes to determine when particular forms of monitoring and control are productive. Second, controlled experiments should compare reciprocal monitoring-control scaffolds with alternative prompting supports, testing whether criterion-based checkpoints before and after adaptation improve coordination and artifact quality without imposing a rigid sequence. Third, studies should directly measure cognitive load and individual differences such as GenAI literacy, trust, and anxiety to evaluate proposed mechanisms of cross-source divergence. Fourth, multisite and stratified studies should examine whether observable monitoring–control relations vary across disciplines, task types, and levels of GenAI experience and acceptance. Finally, age-comparative and longitudinal studies should include younger learners while directly measuring relevant reasoning, metacognitive knowledge, and GenAI literacy rather than inferring developmental stage from chronological age alone. Younger learners may differ in how they externalize criteria, evaluate generated content, and coordinate control with monitoring; these possibilities require direct developmental evidence rather than extrapolation from the present undergraduate sample. The transportability of the coding scheme should also be tested by double-coding participant-level records across educational settings, disciplines, tasks, learner populations, and GenAI systems and configurations, with explicit checks that unitization and category boundaries remain interpretable across contexts.

## Figures and Tables

**Table 1:** Participant Distribution of GenAI Prior Experience and Attitudes (N = 79).

GenAI Usage Experience	Frequency (n)	Percentage (%)	GenAI Usage Attitude	Frequency (n)	Percentage (%)
Never used	16	20.2%	Strongly opposed	1	1.3%
Used, but unfamiliar	27	34.2%	Somewhat opposed	1	1.3%
Moderately familiar	26	32.9%	Neutral	16	20.2%
Relatively familiar	10	12.7%	Somewhat supportive	51	64.5%
Very familiar	0	0.0%	Strongly supportive	10	12.7%

**Table 2:** Operational coding rules for observable metacognitive processes in GenAI-assisted task performance.

Operational Category	Decision Rule	Observable Example
Planning	Specifies a goal, task decomposition, constraint, sequence, anticipated outcome, or resource-selection plan before an action or model response.	Before drafting, help me divide the lesson into inquiry phases and specify the evidence needed to judge each objective.
Monitoring	Expresses a status judgment, comparison, discrepancy, progress check, feedback analysis, or evaluative criterion without yet enacting a change.	The assessment activities do not appear to measure the stated objective; which objective is unsupported?
Regulation	Changes a prompt, strategy, pace, resource, or artifact after feedback or evaluation; the unit must contain an observable revision request or decision.	Because the assessment misses collaborative reasoning, replace the final quiz with an observation rubric and peer-evidence check.

**Table 3:** Decision rules for depth of explicit metacognitive evidence.

Evidence-Depth Label	Observable Decision Rule	Example and Boundary Cue
Implicit	Contains a task request but no articulated goal criterion, rationale, procedure, evaluation, or transfer. The label is used as operational shorthand for underspecified evidence in the unit and does not imply unconscious cognition or the absence of learner awareness.	“Make this lesson better.” A short prompt can be coded higher if it states a criterion; absent wording is not proof of absent awareness.
Awareness	Identifies a goal, issue, uncertainty, or evaluative concern but does not specify how it will be assessed or addressed.	“The objective may be too broad. Is there a problem?” Issue recognition without a criterion or procedure remains awareness.
Strategic	Specifies a task-relevant criterion, decomposition, procedure, or targeted revision tied to the immediate problem.	“Check each activity against the objective and flag any activity without observable evidence.”
Reflective	Evaluates the effectiveness or limitation of an approach and uses that evaluation to justify a change or derive a transferable principle.	“The rubric check found alignment but missed accessibility; add accessibility as a criterion and use it in future lesson reviews.”

**Table 4:** Descriptive distribution of evidence-depth label by operational process category and data source.

Operational Process Category	Evidence-Depth Label	Interaction Log n (%)	Think-Aloud n (%)	Descriptive Higher-Depth Contrast
Planning	Implicit	6 (6.2%)	1 (1.5%)	think-aloud 59.4% vs. logs 49.5%; 9.9 percentage points higher in think-aloud.
	Awareness	43 (44.3%)	27 (39.1%)	
	Strategic	44 (45.4%)	35 (50.7%)	
	Reflective	4 (4.1%)	6 (8.7%)	
Monitoring	Implicit	4 (6.3%)	0 (0.0%)	think-aloud 66.7% vs. logs 41.3%; 25.4 percentage points higher in think-aloud.
	Awareness	33 (52.4%)	16 (33.3%)	
	Strategic	26 (41.3%)	31 (64.6%)	
	Reflective	0 (0.0%)	1 (2.1%)	
Regulation	Implicit	2 (1.8%)	1 (1.3%)	logs 69.3% vs. think-aloud 55.3%; 14.0 percentage points higher in logs.
	Awareness	33 (28.9%)	33 (43.4%)	
	Strategic	76 (66.7%)	40 (52.7%)	
	Reflective	3 (2.6%)	2 (2.6%)	

Note. Percentages are calculated within each operational process category and data source. “Higher-depth” combines strategic and reflective evidence. Differences are descriptive percentage-point contrasts among retained instances. They are not participant-level estimates and have no associated significance tests because instances are nested within participants, sources are paired within individuals, and the available data contain only aggregate coded counts.

## Data Availability

The datasets generated and analysed during the current study are available from the corresponding author on reasonable request.

## Appendix A. Participant Informed Consent & Demographic Materials

### Appendix A.1. Participant Consent Protocol

1. Research Objectives

This study investigates Human–AI collaborative behaviors using the Generative AI platform (DeepSeek). Participants are tasked with completing a pedagogical design scheme, refining the scheme iteratively through multiple engagements with GenAI. The instructional design should encompass various components, such as learner characteristic analysis, establishment of learning objectives, task analysis, preparation of the environment and resources, design of teaching strategies, arrangement of the learning process, selection of evaluation methods, and reflective summarization.

2. Scope of Data Collection
  - Interaction Data: Full logs of prompts and GenAI responses.
  - Cognitive Process Data: Synchronous think-aloud audio recordings.
3. Confidentiality & Rights
  - Anonymization: Data will be processed using numerical identifiers. No personally identifiable information will be disclosed.
  - Storage: Encrypted storage on restricted-access servers; raw data retention for three years post-publication, followed by irreversible deletion.
  - Voluntary Participation: Participation is voluntary. Participants may withdraw without penalty until their data have been irreversibly anonymized and incorporated into the analysis. Before that point, identifiable data contributed by a withdrawing participant will be deleted promptly.

### Appendix A.2. Pre-Task Background Survey

Section 1: Academic Profile

- Academic Discipline/Major: ______________
- Year of Study: ______________

Section 2: Attitudes & Experience

- Q1. Attitude towards GenAI-assisted learning: ______________
  1. Strongly opposed
  2. Somewhat opposed
  3. Neutral
  4. Somewhat supportive
  5. Strongly supportive
- Q2. Prior experience with and familiarity with GenAI tools: ______________
  1. Novice: Never used or used once.
  2. Basic: Used occasionally but unfamiliar with advanced functions.
  3. Intermediate: Moderately familiar with standard prompting.
  4. Advanced: Relatively familiar with prompt refinement.
  5. Expert: Very familiar with complex interaction strategies.

## Appendix B. Experimental Task Instruction Sheet

1. The Scenario

Imagine you are a pre-service teacher preparing for a professional teaching practicum. Your objective is to design a high-quality Instructional Design Plan for a primary or middle school-level topic of your choice (e.g., STEM, Humanities, or Information Technology).

2. Deliverable Requirements

To complete the task, your design must explicitly address the following modules. Collaboration with the AI agent to verify these modules offers an opportunity to demonstrate your planning and regulating strategies.

- Learner Analysis: Definition of target audience characteristics and prior knowledge.
- Instructional Objectives: Specification of measurable cognitive or behavioral goals.
- Content & Activities: Sequencing of pedagogical activities and resource allocation.
- Assessment Scheme: Methods primarily designed to evaluate the learning outcomes.
- Reflective Verification: Logic check of the alignment between objectives and assessment.

3. Human–AI Interaction Rules

- The Constraint: Do NOT simply ask the AI to “write a whole lesson plan for me”. (Such interaction corresponds to the Implicit Level and will be considered invalid task performance).
- The Requirement: You must act as the primary architect. Use the GenAI agent (DeepSeek) as a co-pilot to brainstorm ideas, refine details, challenge its logic, and optimize the flow.

## Appendix C. Think-Aloud Protocol Instructions

Protocol adapted from Ericsson and Simon (1993) to standardize concurrent verbalization procedures.

1. Instruction Script (Read to Participant)

In this experiment, we are studying your cognitive process while collaborating with AI. We ask you to think aloud constantly throughout the entire design session.

- Content: Vocalize specific thoughts passing through your mind. If you are reading the AI’s response, read it aloud. If you are uncertain how to prompt next, speak your hesitation. If you are checking an error such as a bug or hallucination, say it.
- Method: Speak as if you were alone in the room. Do not explain your reasoning to the researcher; simply verbalize the thoughts that pass through your mind while you work.

2. Training Phase (Warm-Up Exercise)

As part of the standardized 10 min training period, participants completed a 5 min warm-up exercise to become familiar with concurrent verbalization while performing a task.

- Prompt: Please visualize the house you lived in during elementary school. Walk through the rooms in your mind to count the windows, and speak your search process aloud while you count.

3. Standardized Researcher Interventions

To minimize observer bias, the researcher intervened only under strict conditions:

- Condition: Silence persisting for >5 s.
- Standard Prompt: Please keep talking.
